# First record of *Alectorobius coniceps* (Ixodoidea: Argasidae) and *Dermacentor* sp. (Ixodoidea: Ixodidae) in Pakistan

**DOI:** 10.3389/fvets.2023.1326734

**Published:** 2024-01-16

**Authors:** Abid Ali, Mehran Khan, Zafar Ullah, Muhammad Numan, Kun-Hsien Tsai, Abdulaziz Alouffi, Mashal M. Almutairi, Tetsuya Tanaka

**Affiliations:** ^1^Department of Zoology, Abdul Wali Khan University Mardan, Mardan, Khyber Pakhtunkhwa, Pakistan; ^2^Department of Zoology, University of Loralai, Loralai, Balochistan, Pakistan; ^3^Institute of Environmental and Occupational Health Sciences, Department of Public Health, College of Public Health, National Taiwan University, Taipei, Taiwan; ^4^King Abdulaziz City for Science and Technology, Riyadh, Saudi Arabia; ^5^Department of Pharmacology and Toxicology, College of Pharmacy, King Saud University, Riyadh, Saudi Arabia; ^6^Laboratory of Infectious Diseases, Joint Faculty of Veterinary Medicine, Kagoshima University, Kagoshima, Japan

**Keywords:** *Alectorobius coniceps*, *Dermacentor* sp., 16S rDNA, *cox1*, Pakistan

## Abstract

*Alectorobius* species are soft ticks primarily infesting birds, such as swallows, while *Dermacentor* species are hard ticks mainly infesting mammals, such as small ruminants. This study for the first time reported on the morphological and molecular bases of two tick species, namely *A. coniceps* and a *Dermacentor* sp. in Pakistan. The former species was examined in swallows’ nests in Khyber Pakhtunkhwa province, while the latter species was examined in small ruminants in Balochistan province. In total, 25 ticks were collected, with 14 ticks morphologically identified as *A. coniceps* (males = 9 and females = 5) and 11 ticks identified as *Dermacentor* sp. (males = 7 and females = 4). Following morphological identification, molecular identification was gained by obtaining 16S rDNA and *cox1* sequences for these ticks. The BLAST results for the 16S rDNA and *cox1* sequences from *A. coniceps* shared a maximum identity of 97.46% and 96.49% with the same species from Malta. The BLAST analysis of the 16S rDNA and *cox1* sequences from *Dermacentor* sp. showed maximum identities of 98.42% and 97.45% with *Dermacentor pavlovskyi* from China. The phylogenetic analysis based on 16S rDNA and *cox1* of *A. coniceps* showed a close evolutionary relationship with the same species. The case of *Dermacentor* sp., based on 16S DNA and *cox1*, indicated a close evolutionary relationship with *Dermacentor pavlovskyi* from China.

## Introduction

Ticks are arthropods that fall under Arachnida and are further categorized into three families: hard ticks (Ixodidae), soft ticks (Argasidae), and Nuttalliellidae (1, 2). With medical significance, they are obligate ectoparasites of semi-terrestrial and terrestrial vertebrates (3–5). Usually, hard ticks take a single extended blood meal during each of their life stages, while soft ticks consume multiple brief blood meals during the nymphal and adult stages (6–8).

The genus *Ornithodoros* is recognized as the most diverse among soft ticks, comprising approximately 130 species (9–11). They have been documented on a wide variety of hosts, including amphibians, birds, mammals, and reptiles (12–16). Previously, this genus was believed to have seven subgenera, including *Alectorobius*, which encompassed ticks such as *Ornithodoros* (*Alectorobius*) *coniceps* (17, 18). However, *Alectorobius* was later reclassified as a distinct genus (19), and this reclassification is adopted in this study. These ticks are distributed worldwide primarily infesting birds and occasionally parasitize humans (6, 20). These parasites have been observed infesting pigeons, ruddy shelducks, swallows, swifts, sparrows, and chickens (6, 13, 20, 21). Throughout their lifespan, they may parasitize a single host or multiple hosts (6, 20). However, they exhibit nidicolous behavior, briefly attaching to their hosts and generally residing in their nests (6, 13, 22). *Alectorobius coniceps* is an ornithophilic species belonging to the mentioned genus, and there are limited morphologically based records of this species from the Oriental region (23).

With approximately 40 species, the *Dermacentor* is the fourth most diverse genus among hard ticks (24). Approximately half of its species are found in the Palearctic region, with the remaining species distributed across Afrotropical, Nearctic, and Oriental regions (24–26). They are primarily three-host ticks, with a prominent preference for mammals, including wild and domestic, and occasionally humans (24, 26, 27). The adult and nymph stages of these parasites have been observed on a range of larger mammals including pigs, deer, antelope, bison, elk, goats, sheep, cattle, camels, horses, and dogs, whereas their larval stages have been found infesting smaller hosts such as rodents and lagomorphs (24, 27). The subgenus *Asiacentor* is mainly found in Asia, and *Dermacentor pavlovskyi* is regarded as the type species for this subgenus (28–31). These ticks have primarily been found infesting small ruminants, such as goats and sheep (29, 31, 32). The known distribution of *D. pavlovskyi* includes Central Asia and China, which are parts of the Palearctic region (26, 29, 31, 32), and there have been no recorded data of this species in the Oriental region.

Currently, there is a lack of consensus on the systematic classification and taxonomy of argasid ticks, including the genus *Alectorobius* (18, 19). Similarly, although the current understanding recognizes seven subgenera within the genus *Dermacentor* (24, 33, 34), the systematics and taxonomy of this genus pose significant challenges (27, 35). In such circumstances, combining morphological and molecular studies on ticks, especially those that are poorly understood, could be crucial for clarifying their phylogenetic position (36–38). Moreover, understanding tick species from different geographic locations is important for shedding light on the evolutionary history of ticks. Although demonstrating characteristics of both the Palearctic and Oriental regions, the knowledge regarding the genera *Alectorobius* and *Dermacentor* is limited within Pakistan. No tick species from the *Alectorobius* genus has been reported, while only *Dermacentor raskemensis* and *Dermacentor marginatus* have been identified morphologically or molecularly within the *Dermacentor* genus in this country (39–43). To address this knowledge gap, in this study, we reported the occurrence and genetic characterization of *Dermacentor* and *Alectorobius* species in Pakistan using morphological and molecular approaches.

## Materials and methods

### Study area

**Figure 1:**
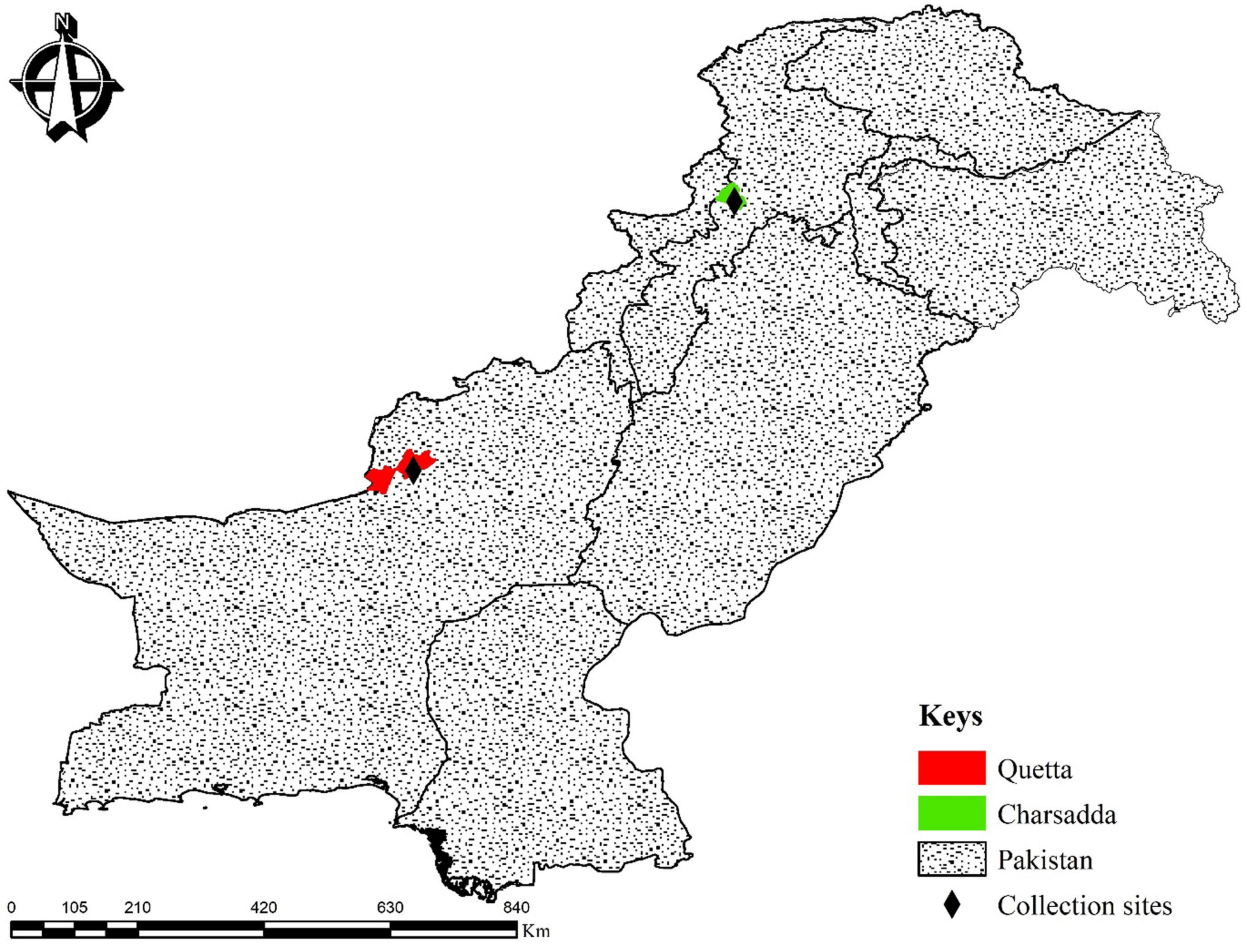
Map showing the collection sites of ticks during this survey.

This study was carried out in the districts of Quetta (30°08′55.3″N, 66°57′42.1″E) and Charsadda (34°10′17.5″N, 71°45′31.7″E) in the provinces of Balochistan and Khyber Pakhtunkhwa, Pakistan, respectively. The geocoordinates of the collection areas were determined using the Global Positioning System (GPS), and the study map was designed using ArcGIS v. 10.3.1 (Figure 1).

### Tick collection and preservation

Tick specimens were collected from sheep and nests of swift birds in 2023 from the districts of Quetta and Charsadda, respectively. In order to avoid any external damage to the specimens, the ticks were carefully detached from the host body and nests using tweezers. The specimens were rinsed in distilled water followed by 70% ethanol and subsequently preserved in 100% ethanol in 1.5 mL Eppendorf tubes.

### Morphological identification of ticks

The collected specimens were morphologically identified using a stereo-zoom microscope (StereoBlue-euromex SB.1302-1, Arnhem, Netherlands) using standard morphological identification keys for *Dermacentor* spp. (28–30) and *Alectorobius* spp. (21, 44, 45).

### DNA extraction and PCR

A total of 14 ticks including five *Dermacentor* spp. and nine *Alectorobius* spp. specimens selected for DNA extraction and were dried in an incubator for 30 min. With the use of sterilized scissors and a micro pestle, the specimens were homogenized in 200 μL of phosphate-buffered saline (PBS). The phenol-chloroform method was used to extract the genomic DNA (46), and 30 μL of “nuclease-free” PCR water was utilized to dilute the extracted DNA pellet. The genomic DNA was measured through NanoDrop (Nano-Q, Optizen, Daejeon, South Korea) and kept at −20°C for further experiments.

Conventional PCR (GE-96G, BIOER, Hangzhou, China) was used to amplify partial fragments of mitochondrial 16S rRNA and *cox1* from the extracted genomic DNA of ticks (Table 1). Each PCR reaction mixture was performed in a total 25 μL volume—containing 1 μL each primer—forward and reverse (10 μM), 2 μL of genomic DNA template (100 ng/μL), 8.5 μL of “nuclease-free” PCR water, and 12.5 μL of DreamTaq green MasterMix (2x; Thermo Scientific, Waltham, MA, United States). A positive control (DNA of *Argas persicus* or *Hyalomma anatolicum*) and a negative control (PCR water that had been “nuclease-free” instead of DNA) were included in each PCR reaction. Lists of the primers used in the present study along with the thermocycler conditions are shown in Table 1.

**Table 1:** List of primers that were used to amplify the ticks’ targeted DNA.

Gene	Primer sequences 5′-3′	Amplicon size	Annealing temperature	References
*cox1*	HC02198: TAAACTTCAGGGTGACCAAAAAATCA	649 bp	95°C 5 min, 40× (95°C 30 s, 48°C 60 s, 72°C 1 min), 72°C 5 min	Folmer et al. (47)
	LCO1490: GGTCAACAAATCATAAAGATATTGG			
16S rDNA	16S + 1: CCGGTCTGAACTCAGATCAAGT	460 bp	95°C 3 min, 40× (95°C 30 s, 56°C 60 s, 72°C 1 min), 72°C 7 min	Mangold et al. (48)
	16S − 1: GCTCAATGATTTTTTAAATTGCTG			

The products of the PCR were electrophoresed on a 2% agarose gel and observed under ultraviolet light in a Gel Documentation System (RB Flash Digi, Robus Technologies, United Kingdom). The DNA Clean and Concentrator Kit (Zymo Research, Irvine, CA, United States) was used to purify the PCR-positive samples in accordance with the instructions provided by the manufacturer.

### DNA sequencing and phylogenetic analysis

All amplified amplicons of *cox1* and 16S rDNA partial fragments were sequenced bidirectionally (Macrogen Inc., Seoul, South Korea) using the Sanger sequencing method. The obtained sequences were cropped through SeqMan v. 5 (DNASTAR, Inc., Madison/WI, United States) to remove poor reading sequences and subjected to Basic Local Alignment Search Tool (BLAST, https://blast.ncbi.nlm.nih.gov/Blast.cgi) at the National Center for Biotechnology Information (NCBI, https://www.ncbi.nlm.nih.gov/). After BLAST, high identity sequences were downloaded in FASTA format from the NCBI. The obtained sequences were aligned with the downloaded sequences using ClustalW multiple alignments in BioEdit Sequence Alignment Editor v. 7.0.5 (49). The phylogenetic trees were constructed individually for each gene sequence of the tick, using the maximum likelihood method with the Tamura-Nei model in Molecular Evolutionary Genetics Analysis (MEGA-X), with a bootstrapping value of 1,000 (50). The coding sequences were aligned using MUSCLE alignments (51).

## Results

### Ticks and their geographic origin

A total of 25 tick specimens were collected and morphologically identified into two distinct species, *A. coniceps* and *Dermacentor* sp., both of which were found in different areas. These comprised 14 out of 25 (56%; 9 males and 5 females) *A. coniceps* from three different nests of swift birds in the district of Charsadda, Khyber Pakhtunkhwa and 11 out of 25 (44%; 7 males and 4 females) *Dermacentor* sp. from sheep in the district of Quetta, Balochistan.

### Morphology of *Alectorobius coniceps*

#### Female

**Figure 2:**
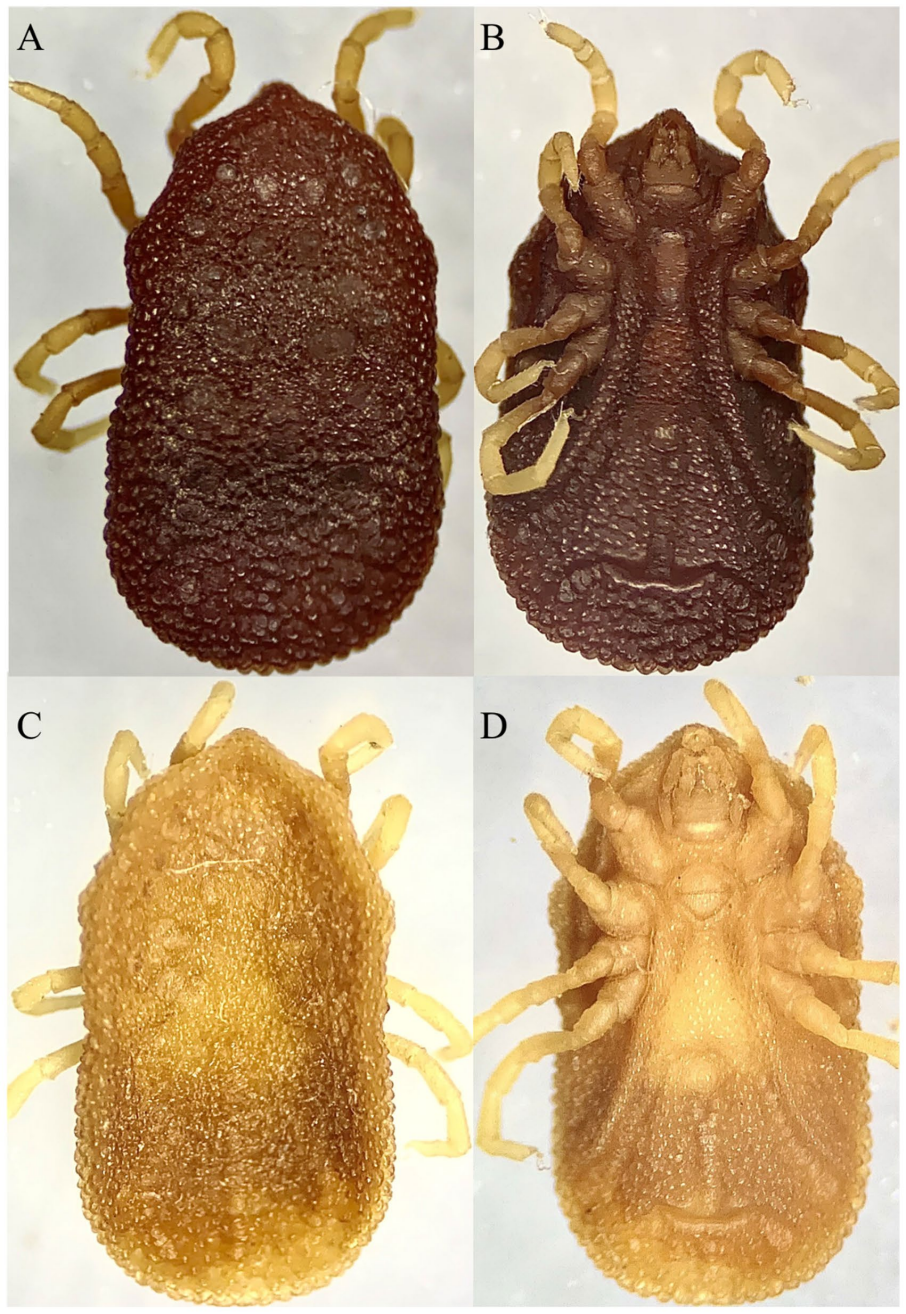
Male (**A**: dorsal and **B**: ventral) and female (**C**: dorsal and **D**: ventral) views of *Alectorobius coniceps* collected in this study from nests of swift birds in district Charsadda, KP.

They are broadly rounded posteriorly, obtusely angled anteriorly with a small, bluntly subtriangular hood, and the color is light to dark brownish to black. Approximately 10 setae and large central pores are found on the anterior labium, and irregularly divided longitudinal striations are found on the posterior labium. The coxal and supracoxal folds are conspicuous. The transverse part of the pre-anal groove is small. With comparatively larger disks adjacent to it, the posteromedian groove extends from the anus to the paired organ. The posterior paired organ can be found at approximately posterior 1 out of 7 to 1 out of 9 of the body. The anterior valve is small and finely striated in shape, whereas the posterior valve is wider and less finely striated in shape with coxa IV disks at each apex. The spiracular plates are large and positioned laterally to coxa IV. The capitulum in the camerostome is between the coxa I and the hood. Basis capituli ventral surface is pebbled and approximately two times as broad as long. Legs are narrow and long, and surfaces are pebbled. Tarsi are narrow, elongate, and abruptly tapering distally (Figure 2).

#### Male

Except for sexual characteristics and size, male ticks are similar to female ticks. The integument around the genital operculum is finely and densely pebbled spiculate, and the posterior integument is transversely rugose. Nymphs resembled adults, except for the lack of external genitalia. Base capituli posthypostomal setae extending to the level of midpalpal segment 2 length. Legs have moderate length and humps (Figure 2).

### Morphology of *Dermacentor* sp.

#### Female

**Figure 3:**
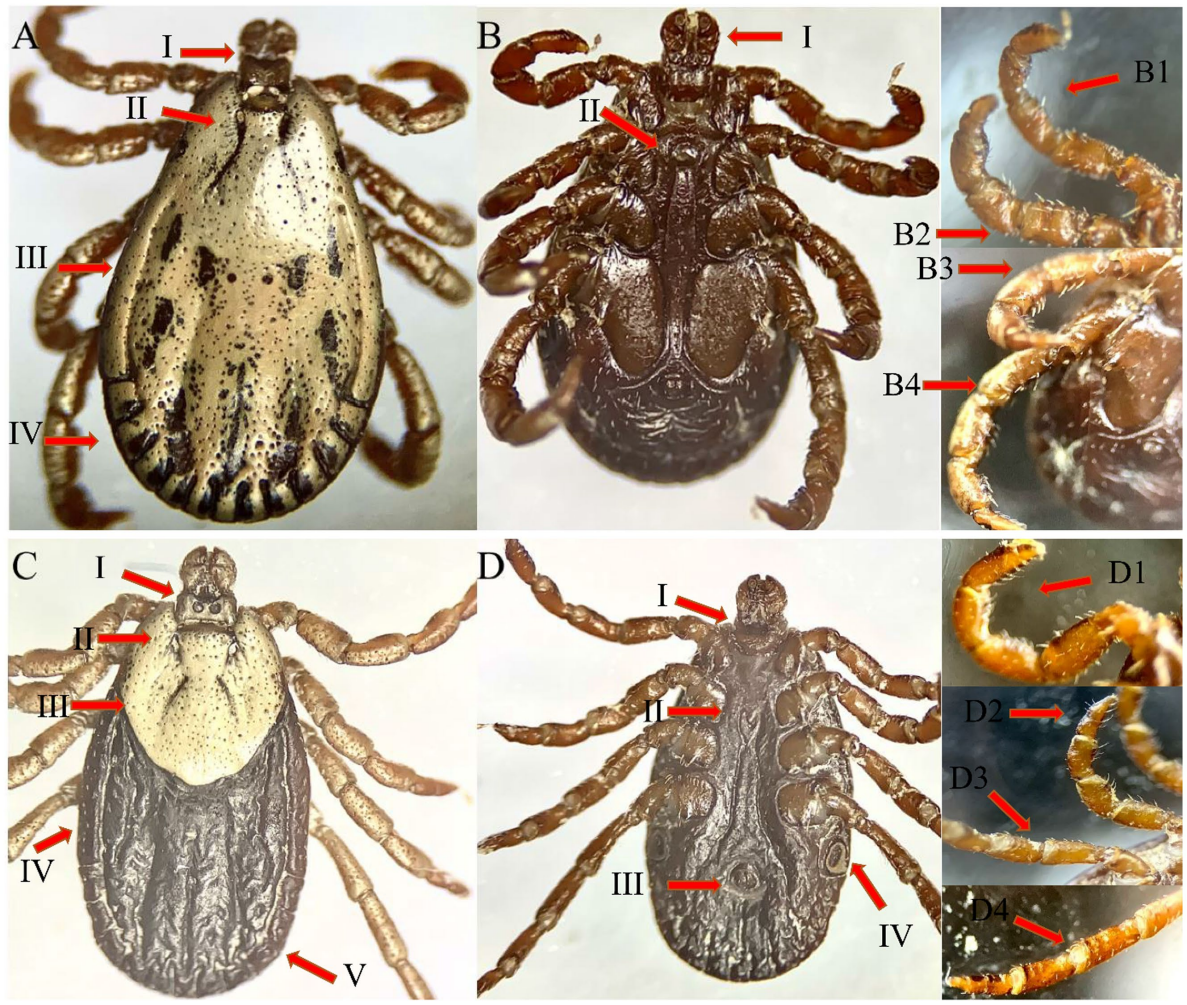
The male [**A**: dorsal—dorsally capitulum (I), cervical groove (II), Lateral groove (III), and festoon (IV), **B**: ventral—ventrally capitulum (I), and genital aperture, leg 1 (B1), leg 2 (B2), leg 3 (B3), leg 4 (B4)] and female [**C**: dorsal—dorsally capitulum (I), cervical groove (II), scutum (III), lateral groove (IV) and festoon (V), **D**: ventral—ventrally capitulum (I), genital aperture (II), anal groove (III) and spiracles (IV), leg 1 (D1), leg 2 (D2), leg 3 (D3), leg 4 (D4)] views of *Dermacentor* sp. collected in this study from district Quetta, Balochistan.

The female tick’s body is elongated, oval, and brownish-red in color. Legs and capitulum are lighter. Except for punctations and grooves, the scutum is oval and whitish in color. Eyes are in front of the middle lateral border. The genital opening is level on coxae II and III. The spiracles are well developed. The capitulum is long and hairy with slightly developed whitish patterns dorsally. Cornua formed barely tubercles, and porose regions are subcircular. Palps are twice as long as they are wide; article I is small; article II and III are both well developed; and article II has a slight posterodorsal spine, while article IV is small and cylindrical. Coxa I has long, triangular, close-spaced, and parallel spurs with tapering or narrowly rounded tips; the external spur is usually nearly equal to or slightly shorter than the internal; and both the spurs are directed slightly posterolateral. Each part of coxae II and III has a sharp external spur and a smaller, broadly oval internal spur; coxa IV is enlarged and rounded with a narrow triangular external spur with a tapering apex. Legs are ornamented, hairy, and lack spines. The tarsi are slightly raised (Figure 3).

#### Male

They are oval and brownish-red in color. The scutum is partially curved, and the whole scutum is white except for the grooves and punctations. The cervical grooves are twisted outward, while the marginal grooves are deep. The outside festoons are wider than the inner ones. The punctations can be seen in some short hairs, some of which are dispersed. Eyes are on a level on coxa II. The genital aperture is parallel to coxa II, and the genital grooves extend almost parallel to coxa IV. They have thin and well-developed spiracles. The cornua is tapered to large spines. The palps are twice as long as the hypostome. Articles II and III are well developed, article II has a tiny spine postero-dorsally, article III is triangular dorsally, and article IV is small and cylindrical. Legs are similar to or resemble those of the female (Figure 3).

### Molecular analysis

**Figure 4:**
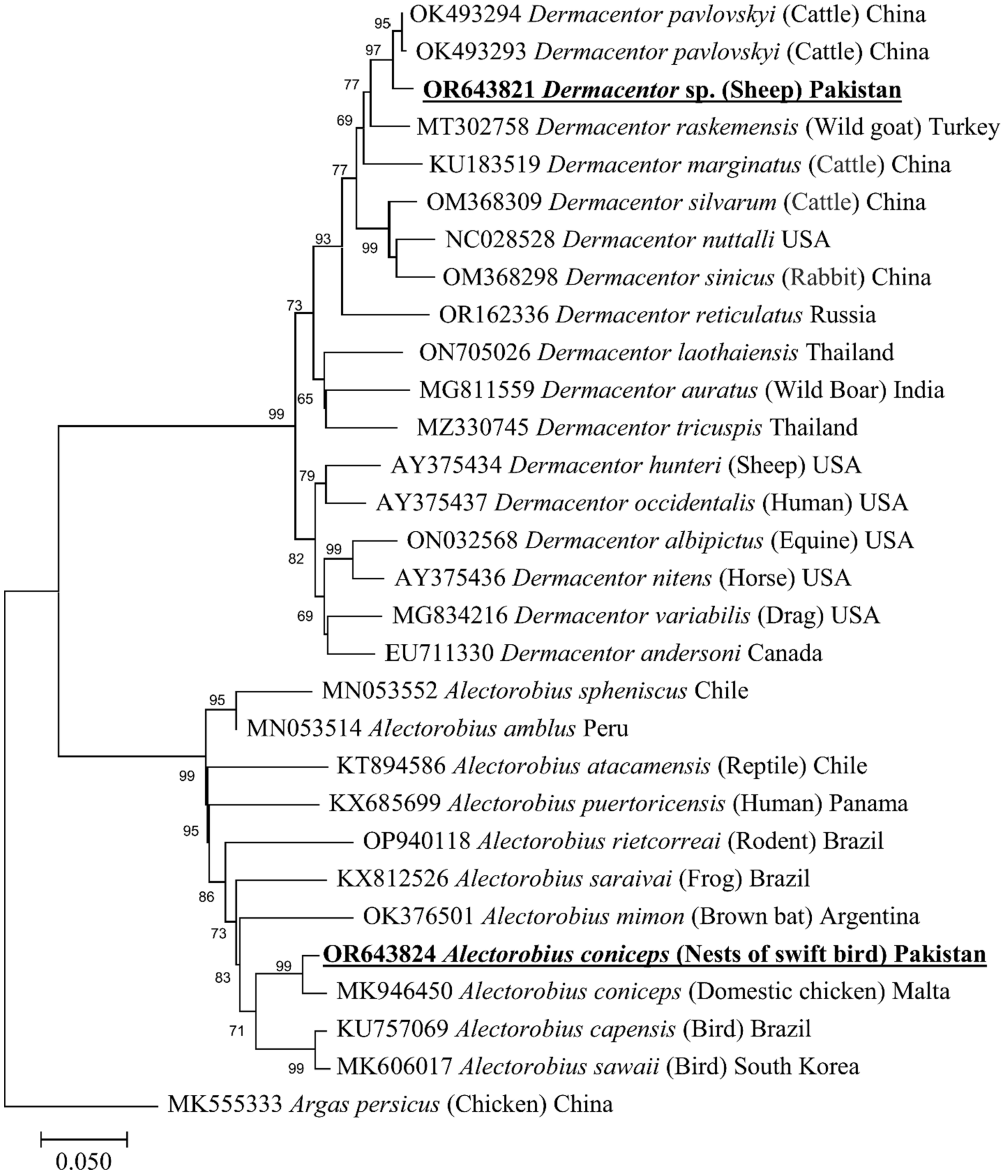
Maximum likelihood phylogenetic tree based on partial mitochondrial 16S ribosomal DNA sequences for *Alectorobius* spp. and *Dermacentor* spp. The 16S rDNA sequence of *Argas persicus* was used as an outgroup. The levels of bootstrap support (>65%) for phylogenetic groupings are given at each node; the accession numbers are followed by the species names, hosts, and locations (if applicable). The obtained sequences are shown in bold-underlined font.

The BLAST analysis of the 16S rDNA sequence belonging to morphologically identified *A. coniceps* showed 97.46% maximum identity with the same species. In the phylogenetic tree, the 16S rDNA sequence for *A. coniceps* was clustered with the same species reported from Malta (MK946450) and grouped in a sister clade with *Alectorobius capensis* (KU946450) and *Alectorobius sawaii* (MK606017). The BLAST analysis of the 16S rDNA sequence for *Dermacentor* sp. showed 98.42% maximum identity with *Dermacentor pavlovskyi*, followed by 96.35% with *Dermacentor marginatus*, 95.79% with *Dermacentor raskemensis*, 95.60% with *Dermacentor niveus*, 94.23% with *Dermacentor nuttalli*, 94.20% with *Dermacentor silvarum*, and 93.70% with *Dermacentor sinicus*. Phylogenetically, the 16S rDNA sequence for *Dermacentor* sp. clustered with the *D. pavlovskyi* reported from China (OK493293-OK493294) and grouped in a sister clade with *D. raskemensis, D. nuttalli, D. marginatus, D. silvarum*, and *D. sinicus* (Figure 4). The obtained 16S rDNA sequence for *A. coniceps* and *Dermacentor* sp. were deposited to the GenBank under accession numbers: OR643824 and OR643821, respectively.

**Figure 5:**
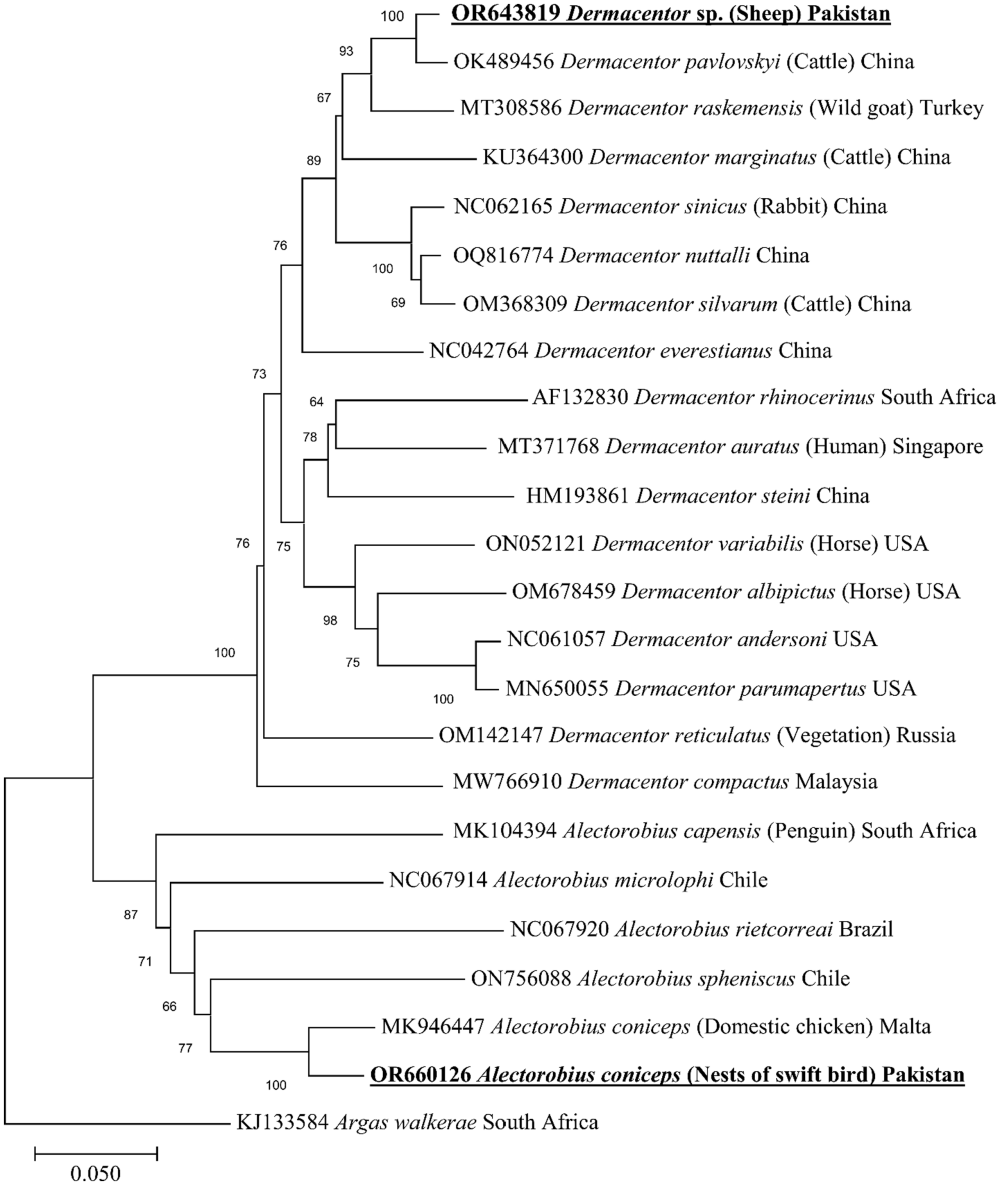
Maximum likelihood phylogenetic tree based on partial mitochondrial *cox1* sequence for *Alectorobius* spp. and *Dermacentor* spp. The *cox1* sequence of *Argas walkerae* was used as an outgroup. The levels of bootstrap support (>65%) for phylogenetic groupings are given at each node; the accession numbers are followed by the species names, hosts, and locations (if applicable). The obtained sequences are shown in bold-underlined font.

The BLAST analysis of the mitochondrial *cox1* partial sequence obtained for *A. coniceps* showed 96.49% maximum identity with the same species. In the phylogenetic tree, the *cox1* sequence for *A. coniceps* clustered with the corresponding sequence was reported from Malta (MK946447). While the BLAST analysis of the obtained *cox1* sequence for *Dermacentor* sp. showed 97.45% maximum identity with *D. pavlovskyi* followed by 93.85% with *D. raskemensis*, 92.09% with *D. nuttalli*, 91.92% with *D. sinicus*, and 91.39% with *D. marginatus*, *D. silvarum*, and *D. niveus.* In the phylogeny, the *cox1* sequence for *Dermacentor* sp. clustered with the *D. pavlovskyi* species was reported from China (OK489456) and grouped in a sister clade with *D. raskemensis*, *D. nuttalli*, *D. sinicus*, *D. marginatus*, and *D. silvarum* (Figure 5). The obtained *cox1* sequences for *A. coniceps* and *Dermacentor* sp. were deposited to the GenBank under accession numbers OR660126 and OR643819, respectively.

The sequences were used in the phylogenetic analysis for *Alectorobius* spp. (Tables 2, 3) and *Dermacentor* spp. (Tables 4, 5), and their identities with the species of the corresponding genus are shown in Table 2.

**Table 2:** Obtained 16S rDNA (OR643824; <460 bp) sequence identities with the diversity of *Alectorobius* species.

Accession numbers	OR643824	MK946450	KU757069	MK606017	KX812526	MN053552	KX685699	KT894586	OK376501	OP940118	MN053514
OR643824	100										
MK946450	97.46	100									
KU757069	92.39	92.39	100								
MK606017	91.83	91.83	98.83	100							
KX812526	89.83	89.83	90.61	90.4	100						
MN053552	88.17	88.17	89.53	89.1	86.35	100					
KX685699	88.03	88.03	87.15	87.34	87.35	85.98	100				
KT894586	87.96	87.96	88.16	88.86	89.37	85.96	87.68	100			
OK376501	87.82	87.82	89.44	89.69	88.32	85.95	85.75	86.92	100		
OP940118	87.43	87.43	88.84	89.04	87.88	85.48	87.38	87.66	86.68	100	
MN053514	87.32	87.32	89.2	89.44	86.35	92.38	86.59	85.71	84.78	87.85	100

**Table 3:** Obtained *cox1* (OR660126; <649 bp) sequence identities with the diversity of *Alectorobius* species.

Accession numbers	OR660126	MK946447	NC067914	MK104394	ON756088	NC067920
OR660126	100					
MK946447	96.49	100				
NC067914	86.48	86.23	100			
MK104394	85.66	83.62	81.34	100		
ON756088	84.98	84.2	77.91	81.4	100	
NC067920	84.04	84.6	78.08	76.07	76.18	100

**Table 4:** Obtained 16S rDNA (OR643821; <460 bp) sequence identities with the diversity of *Dermacentor* species.

Accession numbers	OR643821	OK493294	OK493293	MT302758	KU183519	OM368309	NC028528	OM368298	OR162336	ON705026	MG811559	MZ330745	AY375434	AY375437	ON032568	AY375436	MG834216	EU711330
OR643821	100																	
OK493294	98.42	100																
OK493293	98.16	99.78	100															
MT302758	95.79	96.33	96.11	100														
KU183519	95.31	95.62	95.38	96.59	100													
OM368309	94.2	93.98	93.76	93.35	94.39	100												
NC028528	94.23	94.1	93.79	92.95	93.67	98.82	100											
OM368298	93.7	93.58	93.36	92.95	93.93	97.83	97.63	100										
OR162336	90.98	91.34	91.13	91.4	91.48	85.42	85.29	85.47	100									
ON705026	88.62	88.7	88.46	89.71	89.59	84.3	85.2	85.6	87.3	100								
MG811559	88.39	88.97	88.73	88.52	88.81	85.1	85.29	85.47	86.53	91.77	100							
MZ330745	87.57	89.7	89.47	90.39	89.29	85.2	84.3	85.3	86.6	91.71	91.07	100						
AY375434	86.54	88.94	88.72	88.12	87.02	84.9	83.4	84.55	84.44	89.32	90.31	87.99	100					
AY375437	86.54	88.94	88.72	88.55	87.47	85.42	85.29	85.47	83.2	88.19	89.37	88.66	93.44	100				
ON032568	88.16	88.17	87.96	88.63	88.14	84	81.69	82.08	82.05	88.46	88.73	88.79	92.76	93.67	100			
AY375436	87.89	89.61	89.39	89.44	88.62	84.1	82.5	86.1	85.4	89.35	89.61	89.27	93.89	94.75	95.12	100		
MG834216	88.62	89.83	89.61	89.51	87.98	86.4	85.29	85.47	84.3	89.4	89.88	88.81	94.1	94.53	93.67	94.54	100	
EU711330	87.83	89.08	88.87	89.6	88.41	82.98	83.44	83.4	83.06	90.02	90.19	90.28	93.9	93.7	88.11	94.35	94.99	100

**Table 5:** Obtained *cox1* (OR643819; <649 bp) sequence identities with the diversity of *Dermacentor* species.

Accession numbers	OR643819	OK489456	MT308586	KU364300	OM368309	OQ816774	NC062165	OM142147	ON052121	MT371768	OM678459	NC061057	NC042764	MW766910	HM193861	AF132830	MN650055
OR643819	100																
OK489456	97.45	100															
MT308586	93.85	94.48	100														
KU364300	91.39	92.12	92.14	100													
OM368309	91.39	91.98	92.04	90.49	100												
OQ816774	92.09	92.53	92.71	91.44	86.56	100											
NC062165	91.92	92.14	91.8	91.34	92.3	97.17	100										
OM142147	88.58	88.47	88.66	88.27	88.09	88.04	88.37	100									
ON052121	86.49	87.41	87.59	86.16	83.2	86.69	83.45	86.01	100								
MT371768	86.24	86.62	86.28	85.71	86.39	86.71	86.68	85.17	86.86	100							
OM678459	87.59	87.28	87.01	86.52	82.58	86.35	82.68	86.15	88.36	86.32	100						
NC061057	87.39	87.14	87	85.56	83.52	87.53	83.51	86.01	89.71	86.3	88.49	100					
NC042764	90.16	91.31	90.68	89.53	88.6	90.69	87.07	89.07	83.2	87.22	82.93	83.21	100				
MW766910	85.89	87.03	88.56	85.53	86.95	86.64	86.79	87.3	84.55	86.5	84.91	83.45	84.5	100			
HM193861	86.75	86.79	85.28	84.89	85.58	85.83	86.2	86.1	85.45	87.69	84.31	85.5	85.31	85.19	100		
AF132830	84.34	84.74	84.74	85.13	85.88	84.15	84.72	88.23	85.88	85.29	88.6	85.08	85.55	86.12	85.32	100	
MN650055	87.25	87.09	87.6	84.48	87.6	87.77	87.44	87.7	89.22	85.46	88.7	84.4	85.3	86.02	85.57	85.48	100

## Discussion

Ticks of the genus *Alectorobius* and *Dermacentor* have medical and economic importance worldwide (20, 34, 52). However, they are among the most neglected tick genera in the Oriental region, including Pakistan. Importantly, research in Pakistan has considerably focused on the exploration of ticks and tick-borne pathogens in the last half-decade (4, 5, 9, 39, 53–65). This study provides the first morphological and molecular record of *A. coniceps* and *Dermacentor* sp. from the Oriental region, including Pakistan.

*Alectorobius* spp. are distributed globally possibly due to their association with birds, facilitating an efficient distribution (6, 13, 20). Among *Alectorobius* ticks, *A. coniceps* has been collected from various locations such as caves, crevices, cliffs, ravines, nests, stables, wells, and lofts in both the Palearctic and Oriental regions (20, 21, 66). In this study, *A. coniceps* ticks were collected from swallows’ nests in Khyber Pakhtunkhwa, which is located at the junction of the Palearctic and Oriental regions. Moreover, the larval stages of *Alectorobius* ticks may feed on the same bird species, and therefore, they are relatively well understood from the Palearctic region (13, 21, 67). With this tendency, the adults and nymphs of *A. coniceps* were collected in this study. Future studies should also prioritize the investigation of larval stages from the Oriental region, as, to the best of our knowledge, this stage has not been described in the Oriental region.

*Dermacentor* spp. are believed to have evolved in central Asia, and this region exhibits the highest diversity of *Dermacentor* spp. (26, 68, 69). Among *Dermacentor* ticks, *D. pavlovskyi* has been documented infesting goats and sheep in mountainous regions, as reported in earlier studies (29, 31, 32). Similarly, the area (Balochistan), where *Dermacentor* sp., a closely related tick to *D. pavlovskyi*, was collected in the present study, is situated adjacent to the Palearctic region and has a mountainous terrain with an approximate elevation of 5,500 feet. Other than open areas, *D. pavlovskyi* ticks have been found on wild animals in nature reserves and national parks. For instance, these ticks were collected by the Republican Tropical Station in 1941–1955 in the Aksu-Dzhabagly nature reserve in Kazakhstan (31). Interestingly, the current study area is located near the renowned national park, “Hazarganji-Chiltan.” It could be assumed that *D. pavlovskyi* or closely related ticks have an affinity for wild goats and sheep in such protected areas from where they could invade domestic animals. The tendency of larval *Dermacentor* ticks to infest small animals has resulted in a limited understanding of this stage. Consequently, this study collected adult and nymphal stages of *Dermacentor* sp. ticks, and no larval stage of this species was collected.

Molecular-based analysis is pivotal for comprehending the debated systematics and taxonomy of tick species, including the genus *Alectorobius* and the genus *Dermacentor* (19, 37, 70, 71). Consequently, *A. coniceps* and *Dermacentor* sp. ticks of the current study were subjected to molecular-based analysis involving 16S rDNA and *cox1* sequences. The analysis revealed that the *A. coniceps* ticks from this study displayed variations of 2.54 and 3.51% with their respective species from Malta, based on 16S rDNA and *cox1* sequences, respectively. Similarly, *Dermacentor* sp. ticks exhibited variations of 1.68% and 2.58% with *D. pavlovskyi* from China as determined by 16S rDNA and *cox1* sequences, respectively. At present, although ticks having this range of variations are considered a single species (72, 73, 4), *Dermacentor* sp. could not be validated as *D. pavlovskyi* due to some morphological variations. In contrast to *D. pavlovskyi* and *Dermacentor montanus* (28, 29), *Dermacentor* sp. examined in this study lacks prominent spines on their legs. In comparison to female ticks of *D. montanus* and *D. pavlovskyi*, *Dermacentor* sp. exhibited a genital aperture with a more wing-like shape. Furthermore, other morphological differences among these species were observed in punctuations, cervical grooves, and lateral grooves. However, further comprehensive studies such as mitochondrial genome sequencing of these ticks are essential to gain an accurate understanding of their genetic structure. Furthermore, these intraspecific variations can be attributed to various factors, including tick population size, ecology, and geographic isolation (37, 74). In addition to its closest evolutionary relationship with the same species, *A. coniceps* displayed proximity to *A. capensis*, confirming their classification into the same species complex (6, 13). Although the closest evolutionary relationship of *Dermacentor* sp. with *D. pavlovskyi* was observed, however, its closeness with other species of the same subgenus, such as *D. montanus*, could not be verified due to the lack of authentic genetic data in GenBank.

## Conclusion

This study for the first time presented both morphological and molecular data on poorly known ticks, *A. coniceps*, and *Dermacentor* sp., closely related to *D. pavlovskyi*, from the junction of the Palearctic and Oriental regions in Pakistan. The geographic, morphological, and genetic data of these tick species may aid future studies on tick systematic and taxonomy. Furthermore, the study suggests that the study area, showcasing a combination of traits from two different zoogeographic regions, could harbor a notable diversity of ticks.

## Data availability statement

The data presented in the study have been deposited in the GenBank repository with the following accession numbers: OR643824, OR643821, OR660126, and OR643819.

## Ethics statement

The animal studies were approved by the Advance Studies Research Board (ASRB: Dir/A&R/AWKUM/2022/9396) Committee members of Abdul Wali Khan University, Mardan, KP, Pakistan, gave their approval for the proposed study. The owners of the animals gave their verbal consent for the observation and tick collections. The studies were conducted in accordance with the local legislation and institutional requirements. Written informed consent was obtained from the owners for the participation of their animals in this study.

## Author contributions

AAli: Conceptualization, Funding acquisition, Investigation, Methodology, Project administration, Supervision, Writing – original draft, Writing – review & editing. MK: Formal analysis, Investigation, Methodology, Validation, Visualization, Writing – original draft, Writing – review & editing. ZU: Data curation, Formal analysis, Methodology, Visualization, Writing – original draft. MN: Data curation, Formal analysis, Methodology, Software, Writing – original draft, Writing – review & editing. K-HT: Data curation, Formal analysis, Validation, Writing – review & editing. AAlo: Data curation, Funding acquisition, Investigation, Methodology, Project administration, Writing – original draft, Writing – review & editing. MA: Funding acquisition, Investigation, Methodology, Project administration, Supervision, Writing – original draft, Writing – review & editing. TT: Funding acquisition, Investigation, Methodology, Project administration, Writing – review & editing.
